# *Vaccinium myrtillus* L. ameliorates diabetic nephropathy via modulating metabolites and gut microbiota in rats

**DOI:** 10.3389/fphar.2025.1541947

**Published:** 2025-04-08

**Authors:** Xinxin Cao, Fan Yao, Wenxiu Liu, Yufang Wang, Zhen Zhang, Chongyang Zhang, Zhengqi Dong, Bin Zhang, Ruikun He, Xiaobo Sun

**Affiliations:** ^1^ Institute of Medicinal Plant Development, Peking Union Medical College and Chinese Academy of Medical Sciences, Beijing, China; ^2^ Key Laboratory of Bioactive Substances and Resources Utilization of Chinese Herbal Medicine, Ministry of Education, Beijing, China; ^3^ Diabetes Research Center, Chinese Academy of Medical Sciences, Beijing, China; ^4^ Key Laboratory of efficacy evaluation of Chinese Medicine against Glyeolipid Metabolism Disorder Disease, State Administration of Traditional Chinese Medicine, Beijing, China; ^5^ Innovation Research and Development Center, BY HEALTH Institute of Nutrition & Health, Guangzhou, China

**Keywords:** *Vaccinium myrtillus* L., diabetic nephropathy, gut microbiota, serum metabolites, MAPK/NF-κB

## Abstract

**Introduction:**

Diabetic nephropathy (DN), one of the serious complications in the diabetes, has a high mortality in the diabetic patients. Bilberry (*Vaccinium myrtillus* L.) have received much attention for their health benefits in alleviating metabolic diseases, which are rich in anthocyanins. However, the anti-DN ability of bilberry has not been fully studied. The aim of this study was to investigate the effect and mechanism of *Vaccinium myrtillus* L. extract (VCE) on diabetic nephropathy *in vivo* and *in vitro*.

**Methods:**

Streptozocin (STZ) combined with high fat induced DN model was established in rats. Biochemical indicators, histopathology, 16s third generation sequencing and serum metabolomics were used to evaluate the effects of VCE on DN. Subsequently, a cell model of advanced glycation end products (AGEs) induced podocyte injury was established to verify which compounds in VCE played the main anti-diabetic nephropathy function and the mechanism of action. Finally, *in vitro* experiments were conducted to verify the effect of characteristic metabolites screened by serum metabolomics on improving diabetic nephropathy.

**Results:**

Insulin resistance index, lipid metabolism, oxidative stress and inflammatory response indexes of DN rats were significantly improved after 8 weeks of VCE treatment. In addition, intake of VCE modulates gut microbiota composition and reverses the abundance of *Lactobacillus*, *Bifidobacterium* and *Ruminococcus*. Supplementation with VCE altered serum metabolite levels, including uridine and phenylacetylglycine. Pretreatment with VCE and its anthocyanins inhibited the expression of LDH, IL-6 and TNF-α, reduced the levels of p38-MAPK, IĸBα, IKKβ, and NF-κB in podocyte cells. In addition, pretreatment with serum metabolite uridine also reduced the expression of LDH and mitochondrial ROS, and inhibited cell apoptosis.

**Conclusion:**

Our findings suggest that the improvement of gut microbiota and metabolic function were related to the anti-DN potential of VCE, and the underlying mechanism may be related to the inhibition of MAPK/NF-κB signaling pathway.

## 1 Introduction

The rising global prevalence of diabetes has been increasingly linked to shifts in lifestyle and dietary patterns, with diabetic complications emerging as a critical public health challenge (Zhao et al., 2023). As the most prevalent microvascular complication of diabetes, diabetic nephropathy (DN) represents the primary contributor to diabetes-related morbidity and mortality, accounting for over 40% of end-stage renal disease cases worldwide (Bao et al., 2018). Kidney enlargement and increased glomerular filtration rate are the initial changes in the DN. In patients with advanced DN, the progression from nodular to diffuse glomerulosclerosis and tubulointerstitial fibrosis results in gradual deterioration of renal function (Wang and He, 2021). While the molecular mechanisms underlying DN pathogenesis remain incompletely understood, accumulating evidence implicates oxidative stress-mediated tissue damage and chronic inflammatory responses as central drivers of disease progression (Hanouneh et al., 2021). This mechanistic understanding suggests that targeted interventions mitigating oxidative injury and suppressing inflammatory cascades may offer renal protective benefits and attenuate DN advancement.

Bilberry (*Vaccinium myrtillus* L.), commonly known as European blueberry, is a low-growth shrub native to Northern Europe, but also grown in Asia and North America (Bayazid et al., 2021). Bilberry is rich in anthocyanins, which have a variety of health effects (Dró˙zd˙z et al., 2017; Kylli et al., 2011). Bilberry significantly reduced blood glucose and insulin levels in obese mice, which was associated with a significant increase in glucose transporter 4 (GLUT4) content in skeletal muscle and activation of Adenosine Monophosphate Activated Protein Kinase (AMPK) and Akt pathways (Eid et al., 2014). Bilberry anthocyanin has a protective effect on H9c2 cell apoptosis induced by hydrogen peroxide and can restore the number of surviving cells (Isaak et al., 2017). Bilberry and its anthocyanins (anthocyanin-3-glucoside) could improve plasma lipid and glucose levels and reduce plasma inflammatory cytokine levels in chronic kidney disease, which may be partially mediated through the NF-κB signaling pathway (Hewage et al., 2020). At present, the research mainly focuses on the antioxidant activity of the bilberry, but its prevention of type 2 diabetes and its complications has not been deeply studied.

The gut microbiota constitutes a dynamic microbial ecosystem that critically regulates host physiology through metabolic coordination, immune modulation, and nutrient processing, with dysbiosis increasingly implicated in diabetic pathogenesis (Zhang et al., 2021). Studies have reported that the imbalance of gut microbiota is involved in many links from diabetes to DN, such as insulin secretion disorders, glycation end products, chronic inflammatory state, etc. Identification of gut microbiota can be used as a key biomarker for the diagnosis and prediction of DN (Bakir-gungor et al., 2021; Gurung et al., 2020). Changes in the composition of the gut microbiota affect metabolites. As mediators of the interaction between gut microbiota and disease, metabolites can more directly reflect the relationship between gut microbiota and disease (Tao et al., 2022). The molecular mechanisms of natural products on disease is well revealed by microbiome and metabolomics. Lu et al. (2024) found that the pathogenesis of hypertension and diabetic nephropathy comorbidities was related to Blautia and bile acid metabolites through multi-omics methods (Lu et al., 2024). Based on metabolomics and gut microbiota analysis, Bokhogainsam improved diabetic nephropathy by targeting phospholithin 3-kinase/protein kinase B (PI3K/Akt) and MAPK related proteins (Meng et al., 2020).

Previous studies have reported that bilberry has the potential to regulate gut microbiota in a variety of diseases. The addition of bilberry to a high-fat diet prevents low-grade inflammation and is associated with significant changes in microbiome com-position. The relative abundance of Ackermania and Faecalis increased with increasing intake of bilberry (Lovisa et al., 2016). Bilberry can be used as a preventive dietary measure to optimize the gut microbiome and associated lipid metabolism during and before a high-fat diet, which promotes the production of butyric acid and butyric acid-producing bacteria (Liu H. Y. et al., 2019). Liu et al. (2022) evaluated the metabolic effects of bilberry on gut microbiota and microbe-dependent metabolites and found that both bilberries treated groups had lower Firmicutes/Bacteroidetes ratios and creatinine concentrations (Liu et al., 2022). However, whether bilberry has the ability to regulate gut microbiota and metabolites in DN has not been reported.

In this study, we systematically investigated the protective effects of VCE and its major anthocyanins againstDN using both *in vivo* and *in vitro* models. By integrating 16S rRNA gene sequencing with untargeted serum metabolomics, we sought to unravel the underlying mechanism of VCE-mediated renoprotection through modulation of the gut-serum-kidney metabolic axis. Specifically, our analysis focused on identifying key microbial taxa and differential metabolites associated with VCE treatment that could potentially serve as biomarkers for DN progression. These findings not only advance our understanding of VCE’s therapeutic potential in DN but also provide a rationale for developing VCE-enriched functional foods to prevent or manage this complication.

## 2 Materials and method

### 2.1 Reagents and preparation of extracts

*Vaccinium myrtillus* extract (VCE) is made from the fresh frozen fruit of bilberry (*Vaccinium myrtillus* L.) of the Rhododendron family in Europe and dried after resin refining (Supplementary Figure S1). Delphinidin-3-galactoside chloride (DGA, 68852-84-6), Petunidin-3-O-glucoside (PGL, 6988-81-4), Malvidin-3-O-glucoside (MGL, 7228-78-6), Cyanidin-3-O-arabinoside (CAR, 27214-72-8), Cyanidin-3-O-glucoside (CGL, 7084-24-4) and Cyanidin-3-O-galactoside (CGA, 27661-36-5) (purity >98) were obtained from Sigma-Aldrich (St. Louis, MO, United States). Empagliflozin (Shanghai Yuanye Biotechnology Co. LTD) was used as the positive ctrl in this study.

All cell culture materials, Dulbecco’s modified Eagle’s medium (DMEM, 6124275), fetal bovine serum (FBS, A5256701), and penicillin/streptomycin (15140-122), were obtained from Gibco (Shanghai, China). Advanced glycation end products (AGEs, bs-1158P) were obtained from Beijing Boaosen Biotechnology Co., LTD (Beijing, China) and other chemicals were purchased from Sigma-Aldrich (St. Louis, MO, United States). The low-density lipoprotein (LDL, 100020248); high density lipoprotein (HDL, 100020238); glucose (GLU, 100000240); creatinine (CREA, 100000320); urea nitrogen (UREA, 100020072); β2-microglobulin (β2-MG, 100109070); uric acid (UA, 100020110); urine microalbumin (uALB, 100109060) determination kits were purchased from Zhongsheng Beihang Biotechnology Co., LTD (Beijing, China). Lactate dehydrogenase (LDH, A020-2-2) determination kit was purchased from Nanjing Jiancheng Bioengineering Research Institute Co. LTD (Nanjing, China). N-acetyl-β-D-glucosidase (NAG, 01724R1); kidney injury molecule (KIM-1, 01943R1) ELISA kits were purchased from Jiangsu Jingmei Biotechnology Co., LTD (Jiangsu, China).

Antibodies against p65 NF-κB (10745-1-AP), phospho-p65 NF-κB (82335-1-RR), p38 (14064-1-AP), phospho-p38 (28796-1-AP), IκBα (10268-1-AP) and Iκκβ (15649-1-AP) were purchased from Proteintch (Wuhan, China). And HSP90 (4874) antibody was obtained from Cell Signaling Technology, Inc. (Shanghai, China). Mito-TraCKer Red CMXRos (C1035) and Hoechst 33342 (C1027) kits were purchased from Beyotime Biotechnology (Shanghai, China).

### 2.2 Animals treatments

**FIGURE 1:**
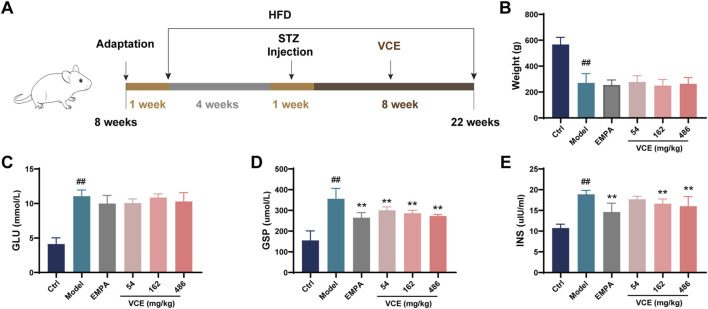
Effects of VCE on glucose and lipid metabolism in diabetic kidney disease rats. **(A)** Experimental design, **(B)** Body weight, Serum **(C)** GLU, **(D)** GSP, **(E)** INS levels in DKD rats. Data are presented as the mean ± SD. #*p* < 0.05 and ##*p* < 0.01 vs. control; *p < 0.05 and **p < 0.01 vs. model. GLU, Glucose; GSP, Glycated serum protein; INS, Insulin; Ctrl, control; EMPA, Empagliflozin; VCE, (*Vaccinium myrtillus* extract).

Male SD rats (250–300 g) were purchased from Beijing Vital River Laboratory Animal Technology Co., Ltd (Beijing, China). A total of 90 rats were used in the experiment. All rats were fed in a standard ctrllable environment for 1 week before the formal experiment, namely, 12 h light/dark cycle, 22°C and 60% in relative humidity (Figure 1A). All experimental animal processing programs were performed in accordance with the Experimental Laboratory Animal Committee of Chinese Academy of Medical Sciences and Peking Union Medical College and performed in accordance with the guidelines of the National Institutes of Health Guide for the Care and Use of Laboratory Animals published by the United States National Institutes of Health (NIH Publication No. 85-23, revised 1996). The protocol number approved by the Research Ethics Committee was SLXD-20220428019. All sacrifices were made under the anesthetic of pentobarbital, and every effort is made to reduce the suffering of the animals.

Two hundred male SD rats were given 50 mg/kg STZ for 1 week after high fat diet (HFD) induction for 4 weeks. Fasting blood glucose was measured at week two. Qualified rats with blood glucose greater than 11.1 mmol/L were divided into 5 groups (n = 15), and 15 normal rats in the same week were selected as blank control group. Specific groups are as follows: (1) SD group (ctrl); (2) STZ-HFD group (Model); (3) STZ-HFD + Empagliflozin 3.5 mg/kg group (EMPA); (4) STZ-HFD + VCE 54 mg/kg group (Low); (5) STZ-HFD + VCE 162 mg/kg group (Intermediate); (6) STZ-HFD + VCE 486 mg/kg group (High). VCE or empagliflozin was fed to the rats by gavage every day for 8 weeks. Rats in ctrl and Model groups were given the same volume of distilled water. All rats were euthanized, rat feces and colon tissues were harvested. Kidney tissues were collected and weighed. One kidney tissue was soaked in paraformaldehyde for pathological analysis, and the other kidney tissue was preserved at −80°C. The organ indices were calculated using the following formula: organ indices (%) = organ weights/body weight (final weights) × 100.

### 2.3 Fasting blood glucose and biochemical marker assays

Fasting blood glucose was measured once a week in the six treatment groups. Fasting blood glucose was measured before intragastric administration or distilled water treatment. Overnight on an empty stomach, the fasting blood glucose in tail venous blood of rats in each group was measured by Roche glucose meter.

The serum concentrations of HDL, LDL, CHOL, LDH, GLU, CREA, UREA and β2-MG were measured with a Mindray BS-420 automatic biochemistry analyzer (Shenzhen, China) following the manufacturer’s instructions. The urine concentrations of UA, uALB, NAG, were also measured with a Mindray BS-420 automatic biochemistry analyzer. ELISA assay kits were used to determine the levels of KIM-1 and β2-MG in urine. ELISA assay kits were used to determine the levels of IL-1β, IL-6, INS, CRP and HO-1 in serum, and activities of GSP, SOD, GSH-PX and MDA in serum using commercial reagent kits (Waldron DR-200BS enzyme label analyzer, Jiangsu, China).

UPLC-MS/MS (equipped with Waters ACQUITY UPLC I-Class AB ultra performance liquid chromatography and SCIEX QTRAP 4500 triple quadrupole tandem linear ion trap mass spectrometry) was used to detect the levels of Nε-carboxymethyllysine (CML) and Nε-carboxyethyllysine (CEL) in plasma. 50 μL of plasma was precisely aspirated, 150 μL of 0.1% formic acid acetonitrile solution containing internal standard (5 ng/ml) was added, vortexed for 5 min, put into a 4°C centrifuge at 1,3000 rpm, centrifuged for 10 min, and the supernatant was quantitatively aspirated for LC-MS/MS detection, and the standard curve was prepared by diluting the standard with aqueous solution.

### 2.4 H&E staining, PAS staining and immunohistochemical assays

H&E staining was performed on colon and kidney tissues as reported previously (Cao X. et al., 2022). PAS staining of kidney tissues was completed according to the method reported by Cao et al. (Cao Y. et al., 2022). Immunohistochemical analyses of nephrin, podocin and 4-Hydroxynonenoic acid (4-HNE) in kidney tissues were conducted as reported previously (Bai et al., 2022; Qi et al., 2020).

### 2.5 Microbiota analysis by 16S rRNA sequencing

Microbial DNA was extracted from fecal samples using the E. Z.N.A.^®^ Soil DNA kit (Omega Bio-tek, Norcross, GA, U.S.). Primers 27F 5′-AGAGTTTGATCMTGGCTCAG-3′ and 1492R 5′-CRGYTACCTTGTTACGACTT-3′ were used to amplify the V1-V9 region of bacterial 16S ribosomal RNA gene. The bar code was a unique 8-base sequence for each sample. The amplicon was extracted from 2% agarose gel. This was then purified using the AxyPrep DNA gel extraction kit (Axygen Biosciences, Union City, CA, U.S.) according to the manufacturer’s instructions.

The SMRTbell library was prepared by blunt tying of amplified DNA according to the manufacturer’s instructions. The SMRTbell library was sequenced in the dedicated PacBio Sequel cell using S/P1-C1.2 sequencing chemistry, this library was purified from Zymo and HMP moCK communities. Purified SMRTbell libraries from pooled and barcoded samples were sequenced on a single PacBio Sequel cell. All amplicon sequencing was completed by Shanghai Biozeron Biotechnology Co. Ltd (Shanghai, China).

### 2.6 Serum metabolomics

Non-targeting metabolic profiling of serums from ctrl (control, n = 6), model (n = 6) and VCE-High (VCE, n = 6), shortly after delivery, were performed using a Vanquish UHPLC system (ThermoFisher, Germany) coupled with an Orbitrap Q Exactive TMHF-X mass spectrometer (Thermo Fisher, Germany) in BIOZERON Biotechnology Co., Ltd. (Shanghai, China).

### 2.7 Cell culture and treatment

The MPC-5 podocytes were purchased from the Shanghai Hongshun Biotechnology Co., LTD (Shanghai, China). The cells were cultured in DMEM medium containing 10% fetal bovine serum, and 1% penicillin/streptomycin at 37°C with 5% CO2. After the cell density reached 80%, the cells were trypsinized and subjected to subsequent experiments.

AGEs, also known as non-enzymatic glycation end products, are stable covalent compounds formed through the reaction between free amino groups of macromolecules such as proteins, lipids, and nucleic acids and aldehyde groups of reducing monosaccharides. Their accumulation *in vivo* plays a critical role in contributing to diabetes and various related complications. Therefore, the age-induced podocyte injury model was first established by using different concentrations of AGEs-induced cells (50, 100, 200 and 400 μg/mL). Subsequently, groups were as follows: (1) Ctrl group; (2) AGEs group; (3) EMPA + AGE group; (4) VCE or Anthocyanin standards + AGEs.

### 2.8 Cell viability, LDH release assays and fluorescent staining

Cells were seeded into 96-well plates at a density of 5 ⅹ 10^4^/well as described in 2.7, and grown for 24 h. Ten microliter CCK8 regent (Dojindo, Japan) was then added to each well. After 2 h of incubation, OD value was measured on a microplate reader at 450 nm (Infinite M1000, Tecan, Sunrise, Austria).

Cell death was assessed by LDH release. The culture medium of podocytes was collected and LDH release was detected by kit according to the manufacturer’s instructions. Fluorescence staining for Mito-TraCKer Red CMXRos and Hoechst 33342 in the podocytes were completed according to the commercial protocol.

### 2.9 Quantitative real-time PCR

Total RNA was extracted by Trizol method (Dong et al., 2020), and cDNA was reverse transcribed using the PrimeScriptTM RT kit (TAKARA, Shiga, Japan). qRT-PCR was performed using SYBR premix Ex Taq II (TAKARA, Shiga, Japan) and then illustrated in a Bio-Rad CFX-96 system. 18S, F 5′- GTAACCCGTTGAACC- CCATT-3′, R 5′-CCA​TCC​AAT​CGG​TAG​TAG​CG-3'; IL-6, F 5′-ATCCAGTTGC- CTTCTTGGGACTGA-3′, R 5′-TAA​GCC​TCC​GAC​TTG​TGA​AGT​GGT-3'; TNF-α, F 5′-AAC​CAG​CAT​TAT​GAG​TCT​C-3′, R 5′- AAC​AAC​TGC​CTT​TAT​ATG​TC-3'.

### 2.10 Western blot analysis

Western blot was performed as described previously (Chen et al., 2020). Podocyte were lysed on ice with RIPA buffer containing protease inhibitors. An equal volume of protein was separated by electrophoresis on 10% SDS-PAGE gels. It was then transferred to 0.45 μm nitrocellulose membrane (Millipore, MA, United States) and left in triglyceride buffer at 300 MA for 1 h. Afterward, the membranes were incubated overnight at 4 °C with appropriate primary antibodies (p65 NF-κB, phospho-p65 NF-κB, p38, phospho-p38, IκBα, Iκκβ and HSP90). After incubation, the membranes were washed with TBST and incubated with secondary antibodies for 2 h at room temperature. The proteins were visualized by enhanced chemiluminescence and analyzed using Image Lab (Bio-Rad, CA, United States).

### 2.11 Statistical analysis

The results were expressed as the mean ± standard deviation (SD). One-way analysis of variance and Tukey’s test were used for comparison between groups (GraphPad Prism 5.0, San Diego, CA, United States). Statistical difference was defined as *p* < 0.01 and *p* < 0.05.

## 3 Results

### 3.1 VCE alleviated the glucose and lipid metabolism in DN rats

From July to September every year, the fresh and mature fruits of *Vaccinium myrtillus* L. were collected, extracted by ethanol, refined by resin, and dried to obtain *Vaccinium myrtillus* L. fruit extract (VCE). HPLC (High Performance Liquid Chromatography) analysis founded that 20 compounds were identified from the VCE, among them, 5 anthocyanins (Delphinidin, Cyanidin, Petunidin, Peonidin and Malvidin), 15 anthocyanins (Cyanidin-3-O-glucoside, Petunidin-3-O-glucoside, Delphinidin-3-O-glucoside, Peonidin-3-O-glucoside, Malvidin-3-O-glucoside, Delphinidin-3-O-galactoside, Cyanidin-3-O-galactoside, Petunidin-3-O-galactoside, Peonidin-3-O-galactoside, Malvidin-3-O-galactoside, Delphinidin-3-O-arabinoside, Cyanidin-3-O-arabinoside, Petunidin-3-O-arabinoside, Peonidin-3-O-arabinoside and Malvidin-3-O-arabinoside) (Supplementary Figure S1).

As shown in Figure 1A, after 4 weeks of high-fat diet (HFD) induction, SD rats were given 50 mg/kg STZ for 1 week. VCE was then administered by gavage for 8 weeks. Through the 8-week VCE intervention, we found that compared with the ctrl group, the body weight of rats in the model group were significantly changed, but there was no significant difference between the VCE treatment group and the model group (Figure 1B).

Glucose (GLU), glycated serum protein (GSP) and insulin (INS) levels were assessed in serum. The effects of VCE on GLU level were not significant compared with model group (Figure 1C). However, the level of GSP in the model group was significantly increased, which was significantly reduced by VCE (54–486 mg/kg) treatment (Figure 1D). The model group enhanced insulin sensitivity, and significantly reduced insulin sensitivity in VCE (162–486 mg/kg) groups compared with model group (Figure 1E). Similarly, compared with the ctrl group, the serum LDL level in the model group were significantly increased. Administration of VCE (54–486 mg/kg) significantly reduced se-rum LDL level compared with the model group (Supplementary Figure S2A). And there was no significant difference in HDL and CHOL levels between the model group and the VCE group (Supplementary Figure S2B, C). These results suggest that VCE might improve glucose and lipid metabolism in DN rats.

### 3.2 VCE alleviated the advanced glycation end products, oxidative stress and inflammatory response in DN rats

**FIGURE 2:**
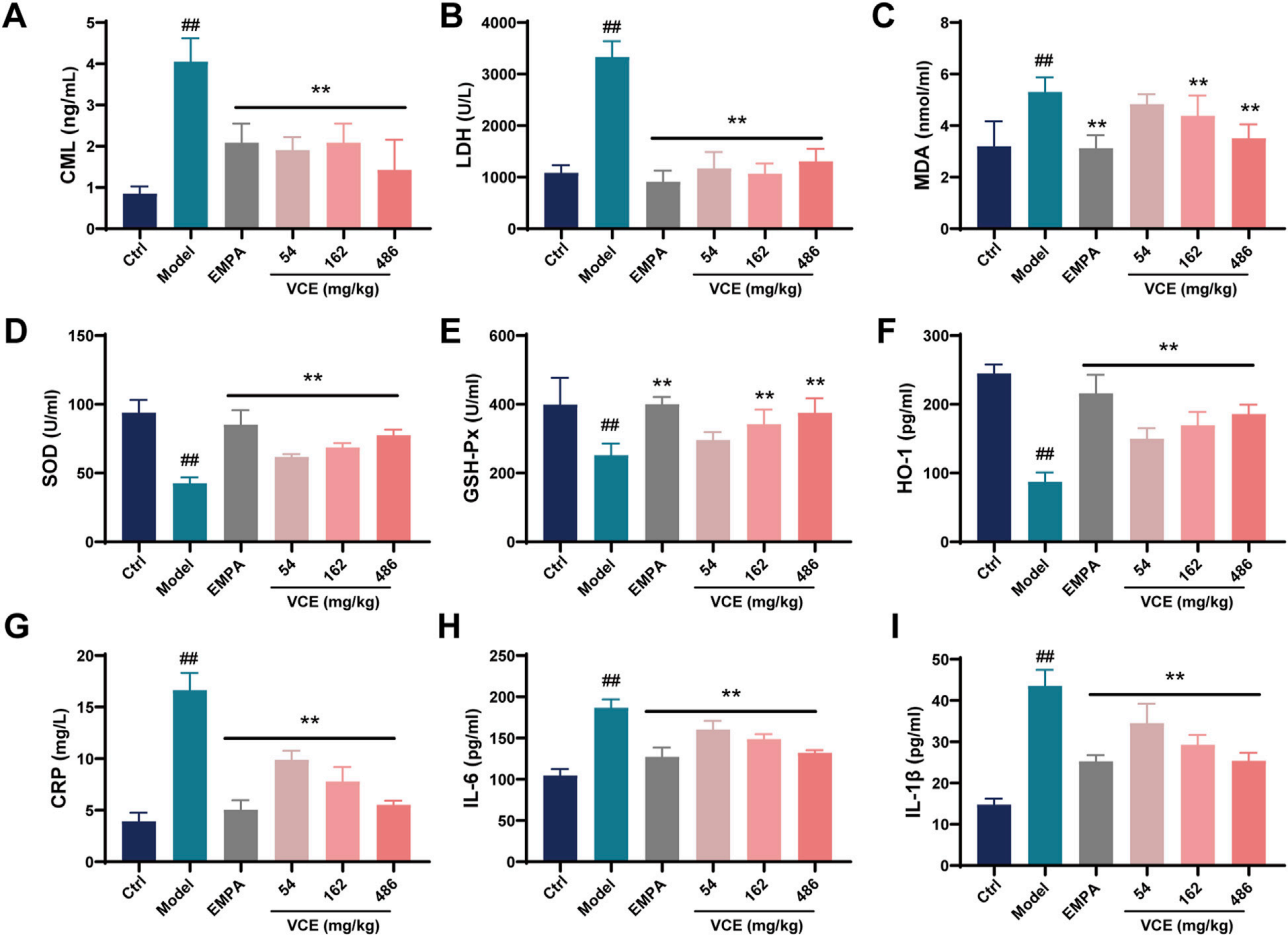
Effects of VCE on advanced glycation end products, oxidative stress and inflammatory response in diabetic kidney disease rats. Serum **(A)** CML, **(B)** LDH, **(C)** MDA, **(D)** SOD, **(E)** GSH-Px, **(F)** HO-1, **(G)** CRP, **(H)** IL-6 and **(I)** IL-1β levels in DKD rats. Data are presented as the mean ± SD. #*p* < 0.05 and ##*p* < 0.01 vs. control; *p < 0.05 and **p < 0.01 vs. model. LDH, Lactate dehydrogenase; MDA, Malondialdehyde; HO-1, Hemeoxygenase-1; SOD, Superoxide dismutase; GSH-PX, Glutathione peroxidase; CRP, C-reactive protein; IL-6, Interleukin-6; IL-1β, Interleukin-1β; CML, Nɛ-carboxymethyllysine; Ctrl, control; EMPA, Empagliflozin; VCE, (*Vaccinium myrtillus* extract).

Advanced glycation end products (AGEs) were involved in oxidative stress, inflammatory response and other pathological changes. At the same time, the occurrence of chronic kidney disease, DN and other diseases is related to AGEs. Nε-carboxymethyllysine (CML) and Nε-carboxyethyllysine (CEL) are two typical AGEs. The model group showed significantly higher CML level compared with the ctrl group. In contrast, VCE treatment significantly reduced high CML level which was significantly lower than that of the model group (Figure 2A). However, there were no significant differences in CEL concentrations between treatment groups (Supplementary Figure S2D).

DN rats had higher LDH and MDA levels compared to the ctrl group, these trends were reversed after VCE treatment (Figures 2B, C). Compared with ctrl group, SOD, GSH-Px and HO-1 levels in model group were decreased significantly. VCE treatment elevated these levels when compared to model group (Figures 2D–F). In the model group, the levels of CRP, IL-6 and IL-1β were prominently elevated relative to the ctrl group. When compared with model group, VCE displayed a reduction in CRP, IL-6, and IL-1β levels (Figures 2G–I). These results suggest that VCE treatment successfully alleviated the HFD and STZ-induced DN in rats.

### 3.3 VCE improved the kidney injury in DN rats

**FIGURE 3:**
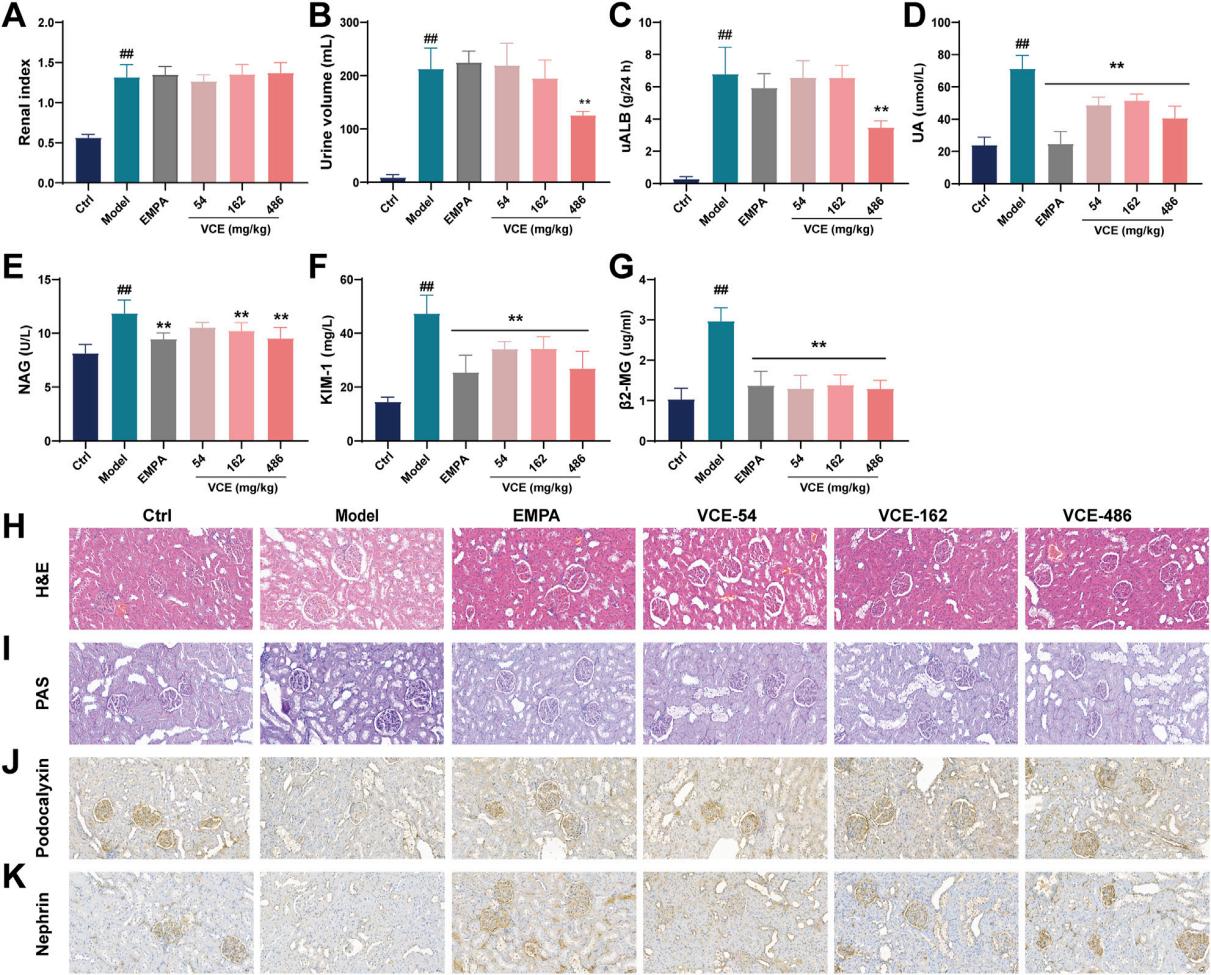
VCE improved renal injury index and renal morphological changes in diabetic kidney disease rats. **(A)** Renal index, **(B)** 24-h urine volume, Urine **(C)** uALB, **(D)** UA, **(E)** NAG, **(F)** KIM-1, **(G)** β2-MG levels in diabetic kidney disease rats. **(H)** H&E staining. **(I)** PAS staining. **(J)** The expression of podocalyxin was detected by Immunofluorescence staining. **(K)** The expression of nephrin was detected by Immunofluorescence staining. Data are presented as the mean ± SD. #*p* < 0.05 and ##*p* < 0.01 vs. control; *p < 0.05 and **p < 0.01 vs. model. uALB, urine microalbumin; UA, Uric acid; NAG, N-acetyl-β-D-glucosidase; KIM-1, Kidney injury molecule; β2-MG, β2-microglobulin; Ctrl, control; EMPA, Empagliflozin; VCE, (*Vaccinium myrtillus* extract). All fields were 200×.

Through the 8-week VCE intervention, we found that compared with the ctrl group, the renal index of rats in the model group were significantly changed, but there was no significant difference between the VCE treatment group and the model group (Figure 3A). The 24 h urine volume and uALB levels in the model group was significantly higher than that in the ctrl group, and they were significantly reduced after high dose of VCE treatment (Figures 3B, C). The UA, NAG, KIM-1 and β2-MG levels in rat urine were enhanced compared with the ctrl group. The levels of UA, KIM and β2-MG were obviously reduced in the VCE-treated groups (Figures 3D, F, G). The levels of NAG were significantly reduced in VCE-162 and VCE-486 treatment groups (Figure 3E). These data indicate that indicators of kidney injury were improved by VCE treatment.

### 3.4 VCE restored histopathological injure in DN rats

We further analyzed the effect of VCE on kidney injury in DN rats at the histological level. Glomerular volume and renal inflammation were examined by H&E staining, and mesangial matrix and mesangial cell proliferation and glomerulosclerosis were examined by PAS staining. Figure 3H showed the H&E histopathological changes of renal tissues of rats. Compared with the ctrl group, the model group showed a series of abnormalities, such as obvious thinning of glomerular basement membrane, and obvious vacuolization of the inner layer of renal tubular epithelium. VCE intervention could im-prove the above lesions, and high-dose VCE (486 mg/kg) has the best effect (Figure 3H). Figure 3I showed PAS staining of rat kidney tissue. Compared with ctrl group, glomeruli and mesangial matrix were significantly enlarged in model group, while the pathological changes of glomeruli were improved in VCE (54–486 mg/kg) treatment groups (Figure 3I). These results indicate that VCE ameliorates the histopathological damage in DN rats.

The expressions of podocalyxin, and nephrin in renal tissues were detected by immunohistochemical staining. The results showed that the expression of podocalyxin and nephrin in the model group were lower than that of the ctrl group, indicating that the renal podocytes of the model group were injured. Relative expression level of podocalyxin and nephrin in the EMPA and VCE groups were higher than that in the model group (Figures 3J, K). These results suggest that VCE may alleviate DN by reversing the expression of nephropathy-related proteins.

### 3.5 VCE regulated the pathological changes of colon tissue in DN rats

**FIGURE 4:**
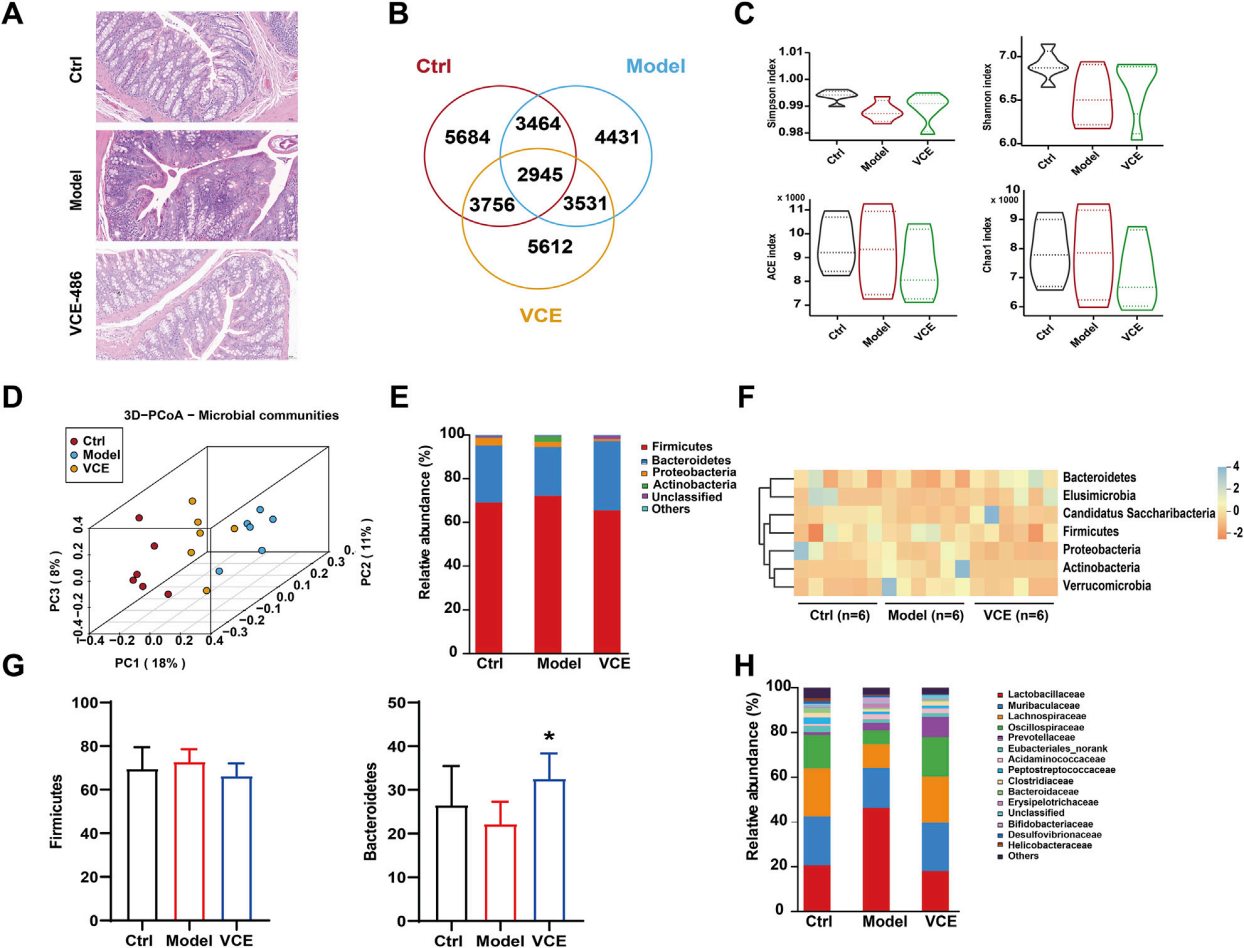
VCE mitigated gut microbiota dysbiosis in diabetic kidney disease rats. **(A)** H&E staining of colon. **(B)** Venn diagram. **(C)** Shannon, Simpson, ACE, and Chao indices. **(D)** PCoA diagram. **(E)** relative abundance of the gut microbiota at the phylum level. **(F)** Hierarchical clustering heatmap of the top differentiated taxa at the phylum level. **(G)** The relative abundance of Firmicutes and Bacteroidetes were compared among groups. **(H)** Relative abundance of the gut microbiota at the family level. The difference between groups was assessed by One-way ANOVA test. *p < 0.05 vs. Model group.

To investigate the intestinal protective effects of VCE in DN rats, H&E staining was performed on colonic tissues from ctrl, model, and high dose VCE (486 mg/kg) treatment groups. Compared with the control group, the model group displayed significant colonic inflammation and tissue damage characterized by crypt loss, leukocyte infiltration, and goblet cell depletion. Notably, VCE administration significantly attenuated these pathological changes (Figure 4A).

### 3.6 VCE regulated gut microbiota at phylum and family levels in DN rats

To further analyze the nephroprotective potential of VCE, fecal samples were sequenced for the 16S rRNA gene sequencing. A total of 4431 microbiotas showed different abundances after HFD and STZ induction, and 3756 microbiotas returned to normal after VCE treatment (Figure 4B), suggesting that microbial of those clusters were potential therapeutic targets for improving DN. No statistical differences were observed between the model and VCE groups in Shannon, Simpson, ACE, and Chao indices (Figure 4C). The cluster of the ctrl group was clearly distinguished from the model group, while the VCE group was partially separated from the model group (Figure 4D), indicating that the overall diversity of gut microbiota changed due to the HFD and STZ-induced diabetes and VCE treatment.

At the phylum level, Firmicutes, Bacteroidetes, Proteobacteria, and Actinobacteriota accounted for more than 90% of the total detected abundance of gut microbiota in all groups (Figure 4E). The seven differentiated taxa with the highest phylum level were shown in the heatmap (Figure 4F). The results indicated that the microbial community composition of the model and ctrl groups had different clustering properties, while most of the VCE treated groups were closer to the ctrl group. We then performed a statistical comparison between groups for the 2 dominance phyla (Figure 4G). The dominant bacterial families in all groups were Lactobacillaceae, Muribaculaceae, Lachnospiraceae and Oscillospiraceae (Figure 4H). These results showed that the composition of gut microbiota was altered in the VCE treated group.

### 3.7 VCE regulated gut microbiota at genus level in DN rats

**FIGURE 5:**
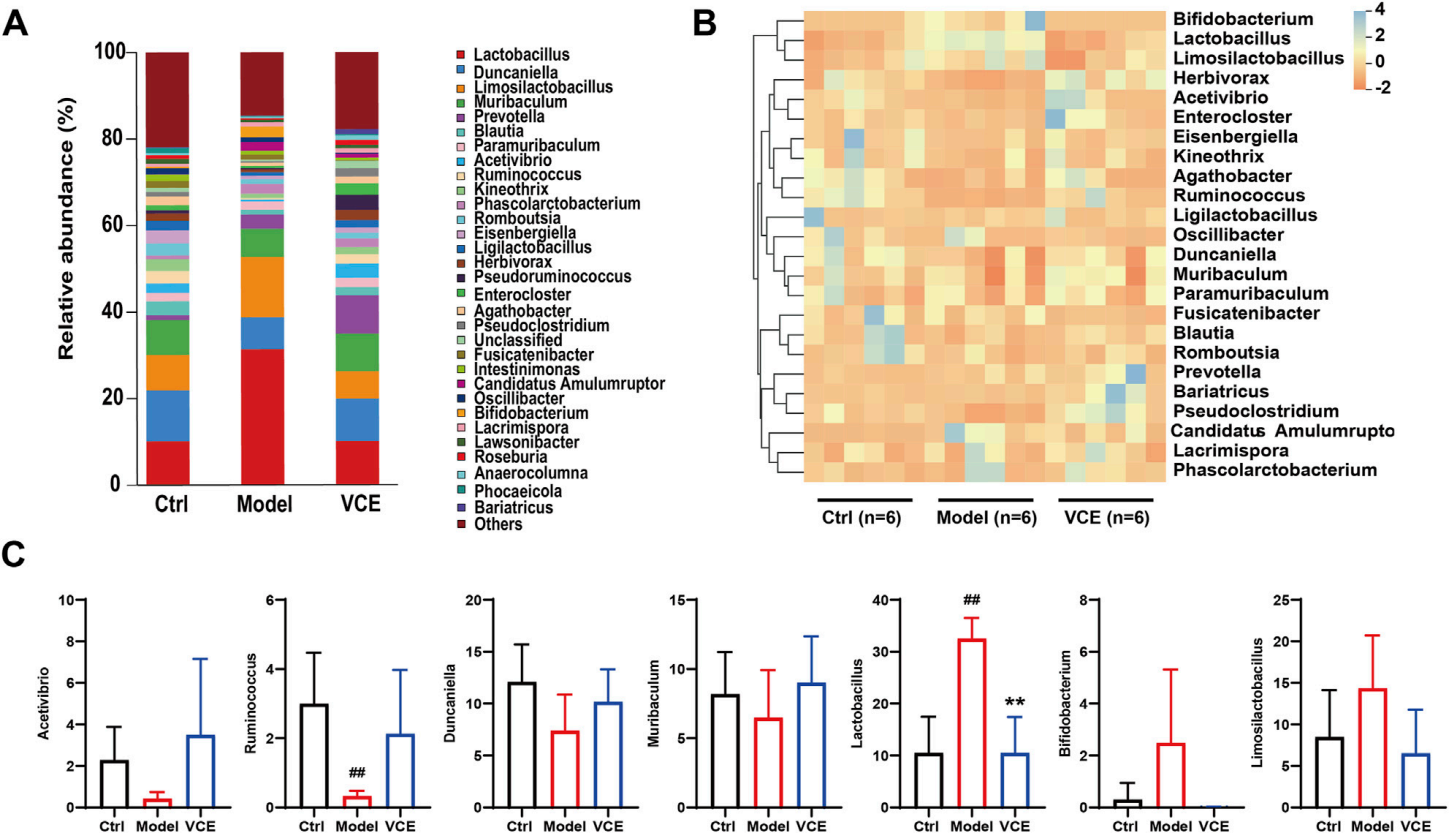
VCE affected the relative abundance of gut microbiota at the genus level. **(A)** relative abundance of the gut microbiota at the genus level. **(B)** Hierarchical clustering heatmap of the top differentiated taxa at the genus level. **(C)** The relative abundance of *Acetivibrio*, *Ruminococcus*, *Duncaniella*, *Muribaculum*, *Lactobacillus*, *Bifidobacterium*, and *Limosilactobacillus*. The difference between groups was assessed by One-way ANOVA test. #*p* < 0.05, ##*p* < 0.01, vs. Control group, *p < 0.05, **p < 0.01, vs. Model group.

Structural changes in the gut microbiota were investigated at the genus level. *Lactobacillus*, *Duncaniella*, *Limosilactobacillus*, *Muribaculum*, *Prevotella*, *Blautia*, *Paramuribaculum* and *Acetivibrio* were the dominant genus in all groups, accounting for about 50% of the total genera (Figure 5A). The heat map demonstrates changes in the relative abundance of dominant microbial genera (Figure 5B). The relative abundance of *Acetivibrio*, *Ruminococcus*, *Duncaniella*, *Muribaculum* were reduced in the model group when compared to the ctrl group (Figure 5C). The relative abundance of *Lactobacillus* in the model group were remarkably high than in the ctrl group, VCE supplementation significantly reduced the abundance of *Lactobacillus* (Figure 5C). *Bifidobacterium* and *Limosilactobacillus* showed an increase in the model groups versus the ctrl groups, but there is no significant difference (Figure 5C). These results suggest that dietary VCE supplementation altered the richness of gut microbiota at the genus level.

### 3.8 VCE regulated serum metabolite profiles in DN rats

**FIGURE 6:**
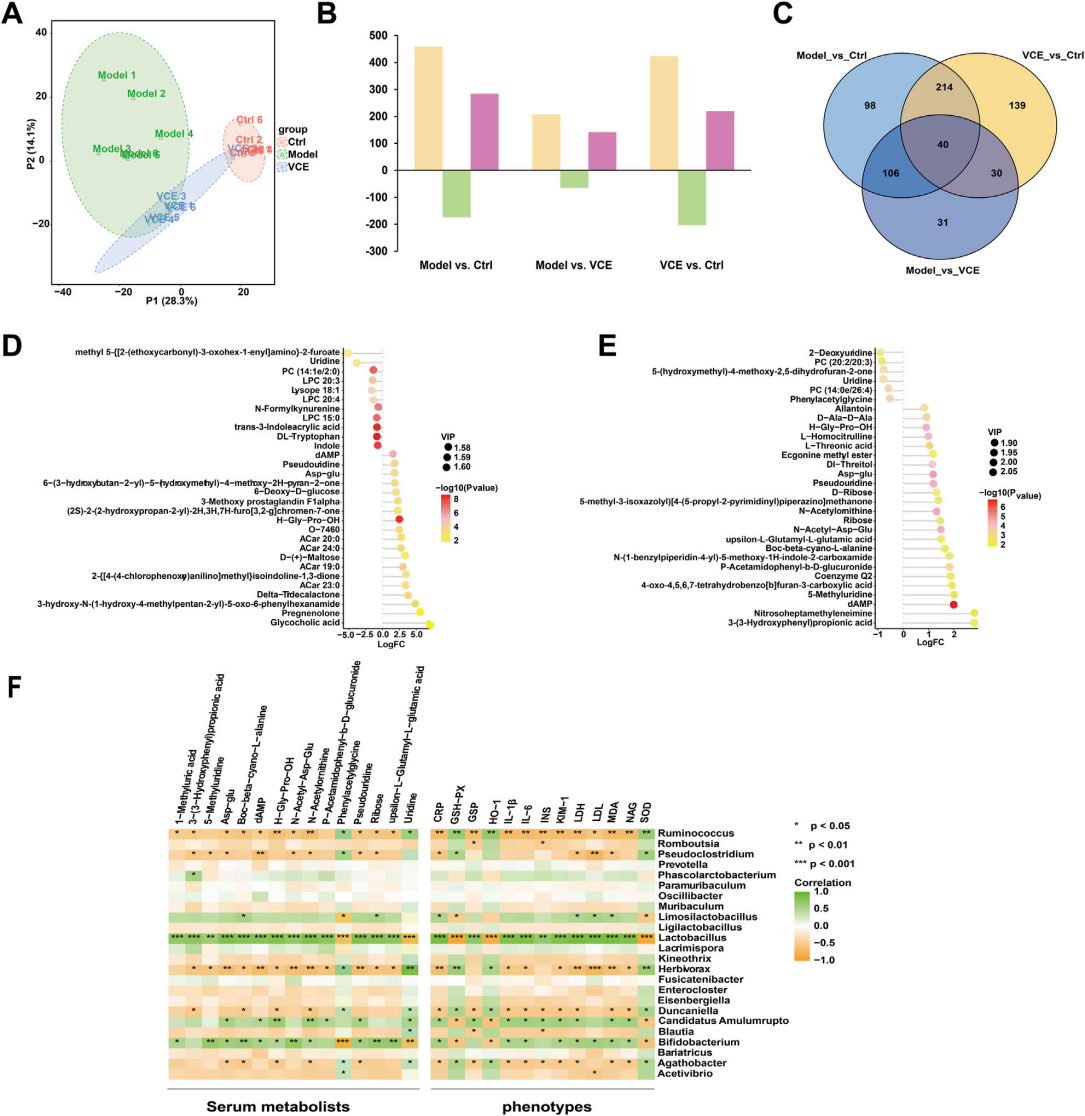
VCE reversed the effects of diabetic kidney disease on serum metabolomic profiles in rat. **(A)** Principal component analysis (PCA) score plots of serum metabolomic profiles in all treatment groups. **(B)** Differentially expressed metabolites identified from pairwise comparisons (yellow, total differentially expressed metabolites; green, downregulated differentially expressed metabolites; rose red, upregulated differentially expressed metabolites. **(C)** Venn diagram of the differentially expressed metabolites identified from pairwise comparisons. **(D)** Lollipop plot of the top 30 of differential metabolites between normal group and model group. **(E)** Lollipop plot of the top 30 of differential metabolites between model group and VCE group. In the figure, the horizontal coordinate represents the multiple of change after logarithmic transformation, the size of the point represents the VIP value, and the color represents the P-value value of the metabolite t-test. **(F)** Spearman correlation analysis of the associations of gut microbiota, serum metabolites, and host phenotypes. P values are shown in gradient colors, with green indicating a positive correlation and brown indicating a negative correlation. Significant correlations are marked by *p < 0.05; **p < 0.01; ***p < 0.001. Ctrl, control; VCE, (*Vaccinium myrtillus* extract).

To investigate the effect of VCE treatment on serum metabolism in rats, an untargeted metabolomic analysis was performed. As shown in Principal Component Analysis (PCA) (Figure 6A), there were differences between the three groups. The ctrl group cluster was separated from the model group. The VCE cluster was partially separated from model and tended to approach ctrl, suggesting that VCE improved the serum metabolite profiles of DN rats. In addition, in paired comparisons, the Orthogonal partial least squares discriminant analysis (OPLS-DA) model was used to distinguish various metabolites, and the model was subsequently judged (Supplementary Figure S3). In total, 458 differential expression metabolites (DEMs) (284 up- and 174 downregulated) were screened in model vs. ctrl, 207 (142 and 65) in model vs. VCE, and 423 (220 and 203) in VCE vs. ctrl (Figure 6B). In addition, 40 DEMs were common to all three comparisons, and 98, 31, and 139 DEMs were unique to the model vs. ctrl, model vs. VCE, and VCE vs. ctrl comparisons, respectively (Figure 6C).

Furthermore, T-test and *p* value were used to examine the significance of metabolites differences between the groups. Figures 6D, E showed the metabolites in the top 30 of the upper and lower of the model vs. ctrl and the model vs. VCE, respectively. Compared with the ctrl group, 11 differential metabolites were downregulated in the model group, including uridine, DL-tryptophan and indole (Figure 6D). As shown in Figure 6E, six differential metabolites were downregulated in the model group compared with the VCE group, including uridine, phenylacetyl glycine, etc (Figure 6E). These results indicated that VCE supplementation might reverse the dysregulation of serum metabolites caused by DN.

### 3.9 Correlations between gut microbiota, serum metabolites, and host phenotypes

Spearman correlation analysis was used to explore potential relationships between gut microbiota at the genus level, serum metabolites, and host phenotypes (Figure 6F). 5-methyluridine, boc-beta-cyano-L-alanine, N-acetyl-asp-glu, ribose and upsilon-L-glutamyl-L-glutamic acid were remarkably and positively correlated with *Lactobacilus* and *Bifidobacterium*. And phenylacetylglycine and uridine were negatively correlated with them. In addition, phenylacetylglycine and uridine was highly and positively correlation with *Ruminococcus*, *Herbivorax* and *Agathobacter*. In the relationship between gut microbiota and host phenotypes, CRP, GSP, IL-1β, IL-6, INS, KIM-1, LDH, LDL, MDA and NAG were significantly and positively correlated with *Lactobacilus*. In contrast, *Ruminococcus* was negatively correlated with the phenotypes described above. CRP, IL-1β, IL-6, KIM-1, LDH, MDA and NAG were positively correlated with *Bifidobacterium* and *Candidatus Amulumruptor*. GSH-Px, HO-1 and SOD were negatively associated with three intestinal bacteria genera, namely *Lactobacilus*, *Bifidobacterium* and *Candidatus Amulumruptor*. In conclusion, serum metabolites and host phenotypes were closely related to the gut microbiota.

### 3.10 VCE and its anthocyanins inhibits AGEs-induced damage in podocyte

To verify the protective effects of VCE against DN *in vitro*, we employed an AGEs-induced podocyte injury model to investigate the cytoprotective properties of VCE and its major anthocyanins. Based on liquid chromatography analysis of VCE (Supplementary Figure S1), six major anthocyanins with high relative abundance were selected for subsequent experiments, including Delphinidin-3-galactoside chloride (DGA), Petunidin-3-O-glucoside (PGL), Malvin-3-O-glucoside (MGL), Cyanidin-3-O-arabinoside (CAR), Cyanidin-3-O-glucoside (CGL) and Cyanidin-3-O-galactoside (CGA).

**FIGURE 7:**
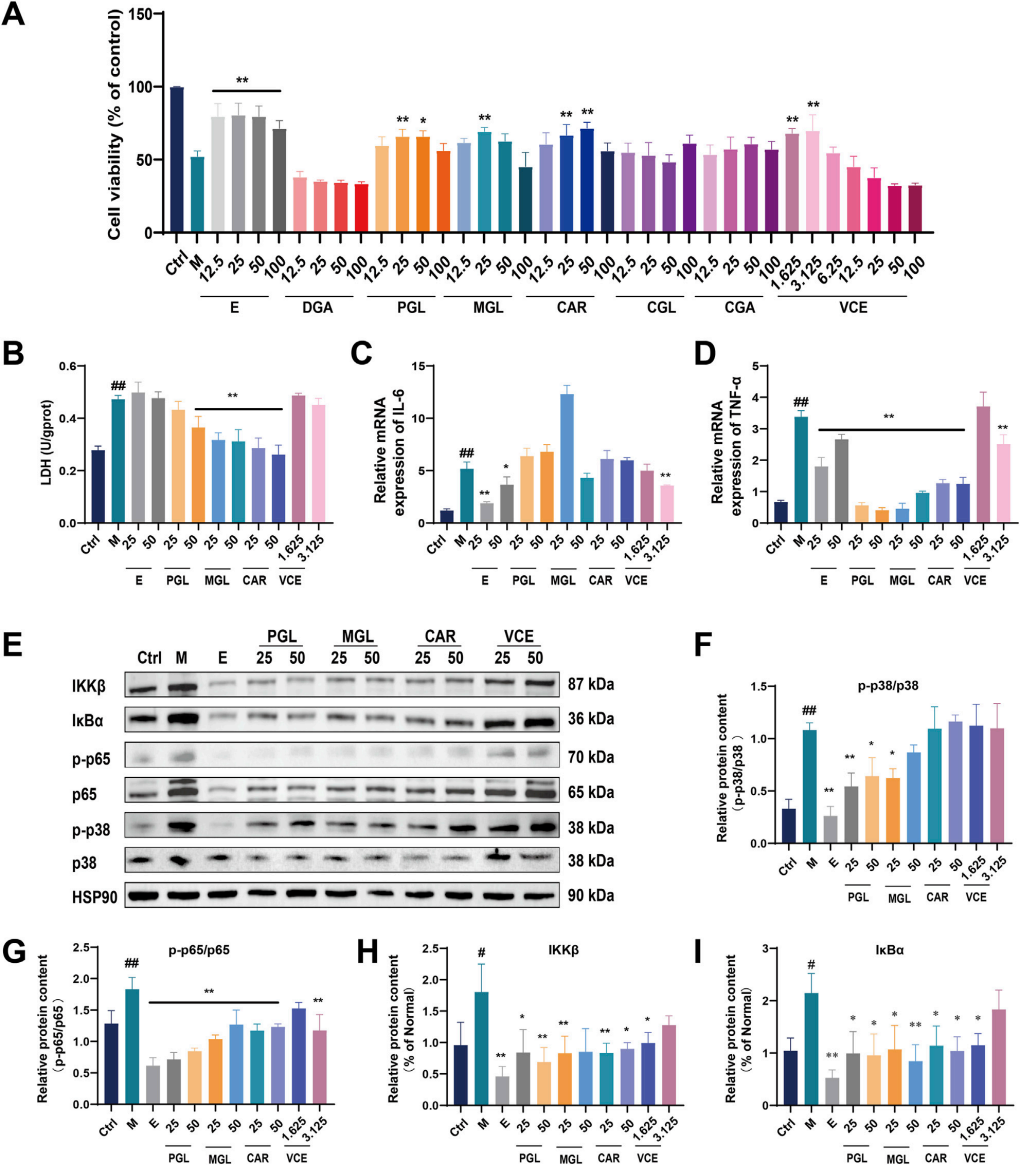
VCE and its anthocyanins attenuated AGEs-induced podocyte injury. **(A)** Cell viability assay. **(B)** LDH detection. **(C)** Quantitative real-time PCR assay for IL-6 and **(D)** TNF-α. **(E)** Western blot analysis of IKKβ, IκBα, p-p65, p65, p-p38 and p38, and HSP90 as a loading ctrl. **(F–I)** Quantification of relative protein expression was performed by densitometric analysis. PGL, Petunidin-3-O-glucoside; MGL, Malvin-3-O-glucoside; CAR, Cyanidin-3-O-arabinoside; Ctrl, control; M, Model; E, Empagliflozin; VCE, *Vaccinium myrtillus* extract. Data are presented as the mean ± SD. #*p* < 0.05 and ##*p* < 0.01 vs. ctrl; *p < 0.05 and **p < 0.01 vs. model.

The cytotoxicity of AGEs was first examined. Podocyte were treated with different concentrations of AGEs (0, 50, 100, 200 and 400 μg/mL) for 24 h. Cell viability was determined by CCK8 assay. As shown in Supplementary Figure S4A, cell viability was reduced to 57.75% ± 0.778% when treated with 200 μg/mL AGEs for 24 h. Therefore, 200 μg/mL AGEs were used in the subsequent experiments (Supplementary Figure S4A). We then evaluated the potential protective effect of VCE and six major chemical components against AGEs-induced injury in podocyte. As shown in Figure 7A and Supplementary Figures S4B–I, cell viability result shows PGL (25, 50 μM), MGL (25 μM), CAR (25, 50 μM), VCE (1.625, 3.125 μM) could reverse the injury on cells caused by AGEs (Figure 7A; Supplementary Figure S4A). Treatment with Empagliflozin was used as a positive control. Lactate dehydrogenase (LDH) leakage was also assessed. Compared with the ctrl group, LDH leakage was significantly increased in the model group, and pretreatment with PGL (50 μM), MGL (25, 50 μM) and CAR (25, 50 μM) effectively reduced LDH release (Figure 7B). These results suggest that PGL, MGL, CAR, and VCE might possess a strong protective effect on AGEs-induced podocyte injury.

### 3.11 VCE and its anthocyanins alleviated AGEs-induced inflammatory response in podocyte

The qRT-PCR was used to detect the protective effect of VCE and three major chemical components on AGEs-induced inflammatory response in podocyte. AGEs treatment significantly increased IL-6 expression compared with the ctrl group; only the VCE (3.125 μM) pretreatment significantly reduced this increase (Figure 7C). As shown in Figure 7D, TNF-α expression was markedly increased in model group compared to those of the ctrl group. The increased TNF-α levels could be conspicuously reversed by administration with PGL (25, 50 μM), MGL (25, 50 μM), CAR (25, 50 μM) and VCE (3.125 μM) when compared with the model group (Figure 7D), indicating that VCE and three major chemical components could protect AGEs-induced inflammatory response in podocyte.

### 3.12 VCE and its anthocyanins alleviated the MAPK/NF-κB signaling pathways in podocyte

In the present study, we detected the expression level of MAPKs family members in podocyte by immunoblotting analysis (Figure 7E; Supplementary Figure S5). The expression of p-p38 and p38 were significantly enhanced in model group. PGL (25, 50 μM), MGL (25 μM) treatment inhibited the activation of the MAPK pathway, which suggested a possible link between anthocyanin of bilberry extract and MAPK activation (Figures 7E, F).

To further evaluate the inhibitory effect of VCE on the inflammatory response, we examined the effect of VCE on NF-κB expression in podocyte using immunoblotting analysis. The protein expression of p65, p-p65, IκBα, and IKKβ were increased in model group. VCE and three major chemical components suppressed the inflammation responses by partial inhibition of the NF-κB signaling pathway (Figure 7E, G–I). Based on the above experimental results, we found that VCE could alleviate AGEs-induced podocyte injury through MAPK/NF-κB signaling pathway, in which PGL, MGL and CAR contribute the main protective effect.

### 3.13 Uridine alleviated the MAPK/NF-κB signaling pathways in podocyte

**FIGURE 8:**
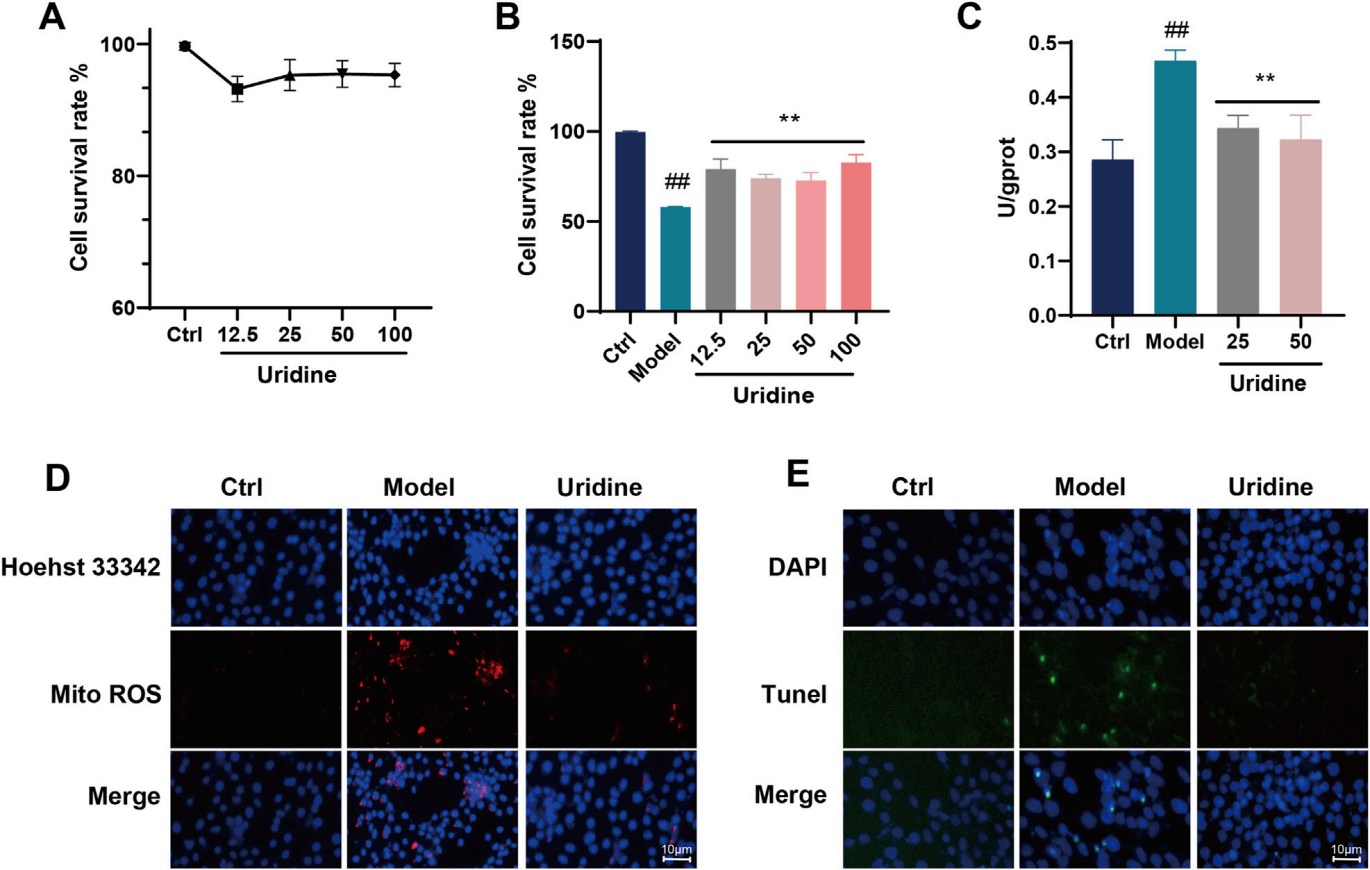
Cytotoxicity assays of **(A)** Uridine. **(B)** Cell viability assay of Uridine against AGEs-induced cell injury. **(C)** LDH assay. **(D)** Mitochondrial ROS fluorescence staining in podocyte cell. **(E)** Tunel fluorescence staining in podocyte cell. Ctrl, control. Data are presented as the mean ± SD. #*p* < 0.05 and ##*p* < 0.01 vs. ctrl; *p < 0.05 and **p < 0.01 vs. model.

The above experiments confirmed the protective effects of VCE and its major anthocyanins against AGEs-induced podocyte injury, but we were still interested in whether the differential metabolites screened by metabolomics also had protective effects against podocyte injury. Therefore, two differential metabolites, uridine and phenylacetylglycine, were selected from serum metabolomics. First, the cytotoxicity of uridine and phenylacetylglycine on podocytes was determined, and it was found that neither of them was toxic to podocytes at the concentration of 12.5–100 μM (Figure 8A; Supplementary Figure S4J). Subsequently, their protective effect on AGEs-induced cell damage was evaluated, and it was found that uridine 12.5–100 μM could reverse AGEs-induced cell damage (Figure 8B), while phenylacetyl glycine had no obvious cytoprotective effect (Supplementary Figure S4K). The effect of uridine on LDH level was then determined. In an AGEs-induced cell model, uridine was found to inhibit LDH release (Figure 8C). In addition, uridine (25 μM) could also inhibit ROS production in cell mitochondria (Figure 8D) and inhibit podocyte apoptosis (Figure 8E). In conclusion, uridine attenuates AGEs-induced podocyte injury by inhibiting oxidative stress and apoptosis.

## 4 Discussion

Diabetes complications are becoming serious health problems worldwide due to high mortality, morbidity, economic burden, and poor prognosis (Yokoyama et al., 2000; Jin et al., 2023; Zhang et al., 2019). Thus, this study systematically evaluated the protective effects of VCE against DN in both STZ-induced diabetic rats and AGEs-treated podocytes. Our results demonstrated that VCE administration significantly ameliorated DN symptoms in rats, as evidenced by reduced levels of key biochemical markers including LDL, INS, and GSP. Mechanistically, VCE treatment was found to attenuate HFD and STZ-induced oxidative stress, inflammatory responses, and intestinal epithelial barrier dysfunction, while concurrently enhancing gut microbial diversity and modulating microbial metabolite profiles. This study shows for the first time that the mechanism of VCE improving DN may be related to regulate intestinal flora, especially Firmicutes, Bacteroidetes, *Lactobacilus*, *Bifidobacterium*, *Ruminococcus*, and metabolite uridine. In addition, *in vitro* experiments verified that VCE may improve DN through MAPK/NF-κB pathway.

In this study, the preventive effect of VCE on DN were explored in diabetic rat and AGEs-treated podocyte. The diabetic rat exhibited typical disordered features including increased levels of INS, GLU, GSP, LDL, LDH as well as renal structural abnormalities. The results presented here proved that VCE could decrease the levels of serum INS, GSP, LDL and LDH levels in diabetic rat, alleviate the pathological changes of the kidney. Li et al. reported that Rg3 significantly reduced serum LDL and MDA levels and increased HDL and SOD levels in HFD/STZ rats (Li et al., 2021). The 3:7 ratio of Astra galus total saponins and Curcumin could significantly reduce the contents of GSP, TG, TC, LDL and MDA, and increase the levels of HDL, SOD and GSH in different degrees in DN rats (Liu B. et al., 2019). GSH, SOD and MDA can be used as indicators to reflect the antioxidant capacity and free radical scavenging capacity of the body (Brezniceanu et al., 2008; Tong et al., 2019). VCE inhibited oxidative stress and inflammatory response by inhibiting the levels of MDA, CRP, IL-6 and IL-1β, and enhancing the levels of SOD, HO-1 and GSH-PX. AGEs are a general term for a series of highly active end products. In diabetic patients, persistently high blood glucose levels in the circulation accelerate the production of AGEs, leading to massive accumulation (Chen et al., 2017). TNF-α and IL-1β are proinflammatory cytokines (Moreno et al., 2018). The mRNA expression level of TNF-α in podocyte cells exposed to AGEs were significantly inhibited by PGL, MGL, CAR and VCE. VCE (3.125 μM) significantly inhibited the mRNA expression level of IL-6 in podocyte cells. These results suggest that VCE has a protective effect on DN *in vitro*. The histological findings (H&E and PAS staining) further confirmed diabetes-related renal damage. Interestingly, VCE treatment improved the histopathological changes caused by DN. Consistent with previous studies, bilberry extract showed the ability to improve diabetic rats (Rocha et al., 2018; Saliani et al., 2023).

Many studies have suggested that intestinal mucosal barrier is related to the regulation of intestinal flora, especially the recovery of probiotics is an important mechanism for its fight against DN (Brunkwall and Orho-melander, 2017; Li et al., 2017; Wen and Duffy, 2017). Our results showed that VCE significantly reversed the increase in *Lactobacillus* and *Bifidobacteria* in the DN. This may help to relieve hyperglycemia, reduce lipid accumulation and tissue damage in the DN rats. The genus *Lactobacillus* could cause an imbalance between Firmicutes and Bacteroidetes. Firmicutes and Bacteroidetes were the two most important phyla associated with the homeostasis of energy metabolism in the gastrointestinal tract (Turnbaugh et al., 2005; Rinninella et al., 2019; Lecamwasam et al., 2020). A randomized double-blind controlled trial showed that probiotic yogurt containing *Lactobacillus acidophilus* La5 and *Bifidobacterium lactis* Bb12 increased total and LDL cholesterol levels in patients with T2D (Ejtahed et al., 2011). Some studies have shown that the increase of *Lachnospiraceae*_ NK4A136_group, *Bifidobacterium* and *Lactobacillus* may be one of the reasons for the disorder of glucose metabolism (Huang et al., 2023). In addition, the VCE treated group increased the relative abundance of *Ruminococcus* in the present study. Studies have confirmed that in T2DM, Lycium barbarum polysaccharide treatment could increase the relative abundance of *Bacteroides*, *Ruminococcaceae*_UCG-014, *Enterinimonas*, *Mucispirillum* and *Ruminococcaceae*_UCG-009 (Ma et al., 2022). *Ruminococcus* typically responds to the treatment of Chlorella pyrenoidosa in rats, thus it may play an important role in the treatment of diabetes (Wan et al., 2019). In ZuCKer diabetic fatty rats, random blood glucose levels were positively correlated with *Ruminococcus* (Zhou et al., 2019). Capsaicin enhanced changes in the relative abundance of key microorganisms such as *Ruminococcus*, *Prevotella*, *Allobaculum*, *Sutterella*, and *Oscillospira*, and the effects of these genera on the gut-brain axis were also associated with inhibition of LPS and TNF-α levels (Xia et al., 2021). In line with the above studies, the VCE significantly reduced *Bifidobacteria* and *Lactobacillus*, and increased the abundance of *Ruminococcus*. Accordingly, VCE supplementation is beneficial to change intestinal flora and may help alleviate glucose and lipid metabolism disorders in DN rats.

Serum metabolomics analysis showed that the disturbed serum metabolic profile could be improved by VCE. Uridine, one of the four components that make up RNA, has attracted attention as a novel therapeutic regulator of inflammation (Nucleosides et al., 2016). Studies have shown that the levels of proinflammatory cytokines IL-6, IL-1β, TNF-α and mRNA expression in colon were decreased in uridine treatment group, which may be related to the inhibition of neutrophil infiltration and NF-κB signal transduction (Jeengar et al., 2017). Another study showed that uridine, a precursor of mitoATP activator UDP, was protective against hypoxic injury in the lung. Uridine also protected the epithelial, interstitial, and inner layers of the air-blood barrier from hypoxia-induced overhydration (Rozova et al., 2019). In this study, uridine was remarkably and negatively correlated with *Lactobacilus* and *Bifidobacterium*. These bacteria were positively correlated with kidney injury index, such as CRP, GSP, IL-1β, IL-6, INS, KIM-1, LDH, LDL, MDA and NAG. Therefore, uridine may be involved in the relief of symptoms related to DN. A study confirmed that four endogenous metabolites, phenylacetylglycine, creatinine, hippuric acid and gluconic acid, could be used as urine biomarkers in diabetic rats by quantitative analysis (Pi et al., 2014). Sequential administration of phenylacetylglycine at an appropriate dose inhibited apoptosis and reduced infarct size in mice induced by I/R injury (Xu et al., 2021). In the present study, the content of phenylacetylglycine decreased after VCE treatment in DN rats, indicating that phenylacetylglycine may be involved in reducing the damage of DN.

In addition to glucose and lipid metabolism disorders, experimental and clinical data have confirmed that oxidative stress and inflammatory response is a key factor in the development of DN (Ni et al., 2019). To further validate these findings, we examined the effects of VCE and its major anthocyanins on AGEs-stimulated podocytes. Quantitative real-time PCR analysis revealed significant upregulation of inflammatory cytokine mRNA (TNF-α and IL-6) in AGE-treated cell. Notably, this pro-inflammatory response was dose-dependently attenuated by VCE and its three major anthocyanins: Petunidin-3-O-glucoside, Malvidin-3-O-glucoside, and Cyanidin-3-O-arabinoside. NF-κB, an important transcription factor that regulates immune response and cytokine expression, was also involved in the progression of DN (Tamada et al., 2006; Hou et al., 2017). In this experiment, the expression levels of p-p65, p65, IκBα and IKKβ were increased in model group. Treatment with VCE and the three major anthocyanins significantly reversed the increased levels of these proteins. As one of the subfamilies of MAPKs, p38-MAPK plays an important role in renal cytokine-mediated inflammation, cell hypertrophy and renal fibrosis (Kyriakis and Avruch, 2024). Because MAPKs activate the release of TNF-α and IL-6 (Kim et al., 2011). The levels of p-p38 and p38 were significantly increased in the AGEs treatment group and decreased after pretreatment with VCE and the three major anthocyanins. These results further confirmed that VCE and the three major anthocyanins reduce the inflammatory stimulation of podocytes by inhibiting the MAPK/NF-κB signal pathway.

**FIGURE 9:**
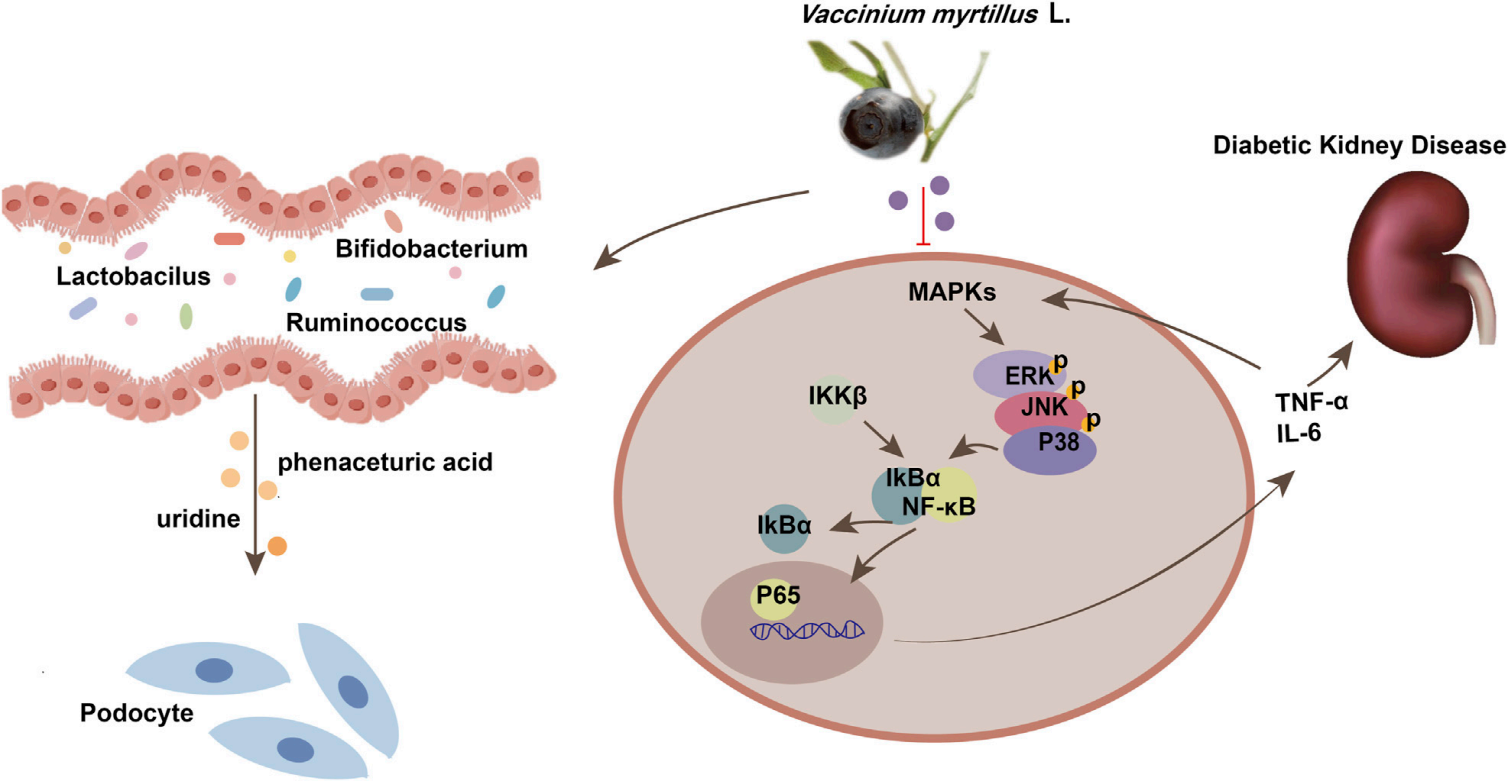
Schematic diagram of the mechanism of MAPK/NF-κB pathway in diabetic kidney disease. MAPKs, Mitogen Activated Protein Kinases; TNF-α, tumor necrosis factor; IL-6, Interleukin-6.

In conclusion, VCE treatment could reduce oxidative stress and inflammatory response in DN (Figure 9). Meanwhile, it restored the integrity of the intestinal barrier and reduced circulating proinflammatory mediators, including TNF-α and IL-6. The decrease of inflammatory response may be related to the regulation of gut microbiota. VCE improved overall diversity, corrected Firmicutes and Bacteroidetes at the phylum level, The relative abundance of *Lactobacillus*, *Bifidobacterium*, and *Ruminococcus* was corrected at the genus level. In addition, VCE also reversed the levels of uridine and phenylacetylglycine. Uridine inhibited AGEs-induced podocyte injury *in vitro*. In combination with *in vitro* and *in vivo* experiments, the protective effect of VCE on DN was at least partially mediated by the regulation of MAPK/NF-κB pathway. Three major anthocyanins in VCE may play key anti-inflammatory roles, including Petunidin-3-O-glucoside, Malvin-3-O-glucoside, and Cyanidin-3-O-arabinoside. Based on these results, we propose that VCE-rich dietary interventions may be effective in delaying the progression of DN in patients. These studies may contribute to clinical trials of VCE in the treatment of DN.

## Data Availability

The microbiome data presented in the study are deposited in the Genome Sequence Archive of the Beijing institute of Genomics BIG Data Center, Chinese Academy of Sciences, accession number CRA023972.
