# Supplementary material for: Vaccinium myrtillus L. ameliorates diabetic nephropathy via modulating metabolites and gut microbiota in rats
Source: Front Pharmacol. 2025 Apr 8;16:1541947. doi: 10.3389/fphar.2025.1541947 (PMC12011793; doi:10.3389/fphar.2025.1541947)
Supplement: Supplementary file 1 [file DataSheet1.pdf]

Figure S1-5

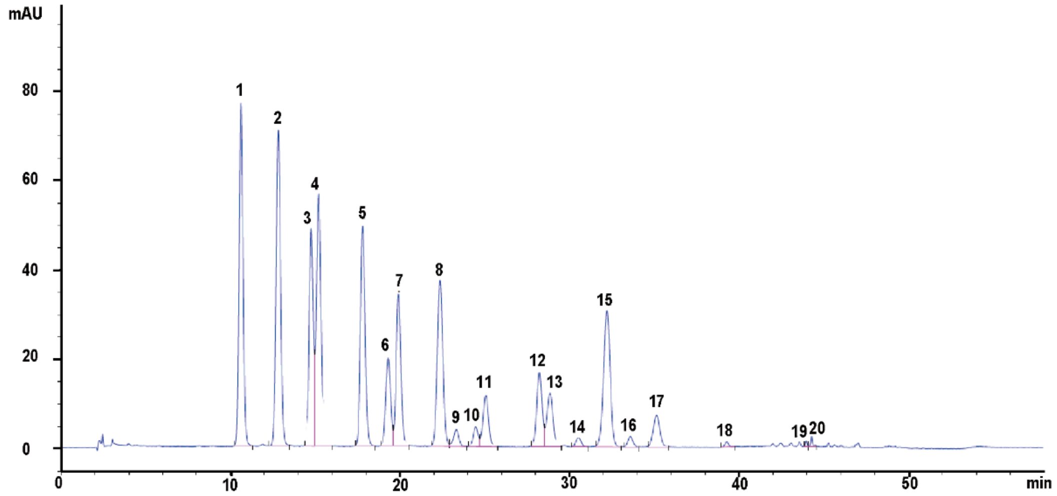

1	Delphinidin - 3-O-galactoside	11	Petunin-3-O-arabinoside
2	Delphinidin - 3-O-glucoside	12	Paeoniflorin-3-O-glucoside
3	Cyanidin - 3-O-galactoside	13	Malvacin-3-O-galactoside
4	Delphinidin - 3-O-arabinoside	14	Paeoniflorin-3-O-arabinoside
5	Cyanidin - 3-O-glucoside	15	Malvin-3-O-glucoside
6	Petunin-3-O-galactoside	16	Cyanidin
7	Cyanidin - 3-O-arabinoside	17	Malvin-3-O-arabinoside
8	Petunin-3-O-glucoside	18	Petunidin
9	Delphinidin	19	Peonidin
10	Paeoniflorin-3-O-galactoside	20	Malvidin

Figure S1 High Performance Liquid Chromatography of *Vaccinium myrtillus* L. extract.

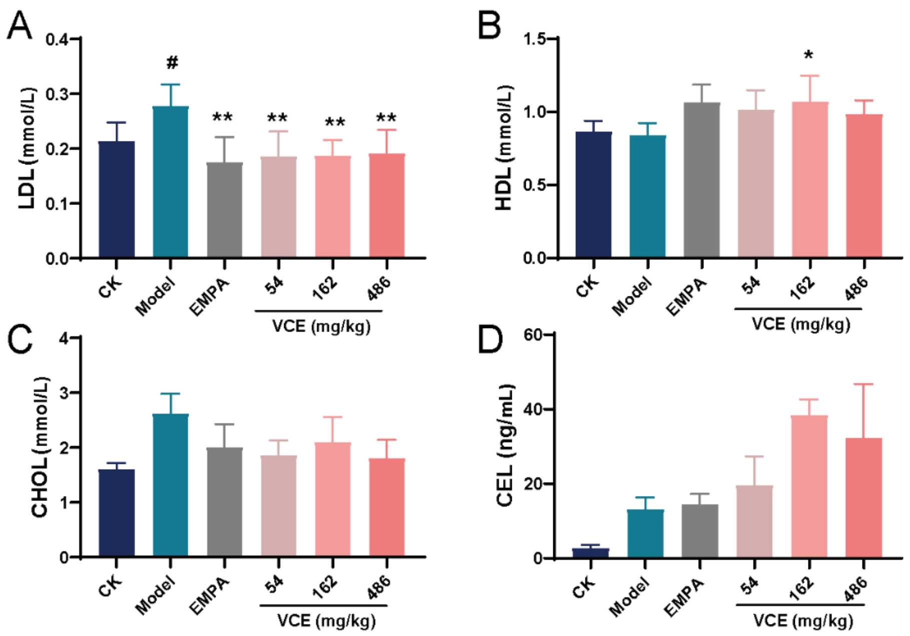

Figure S2 Effects of VCE on blood lipids and advanced glycation end products in diabetic kidney disease rats. (A) Low density lipoprotein, LDL. (B) High density lipoprotein, HDL. (C) Cholesterol, CHOL. (D) Nε-carboxyethyllysine, CEL.

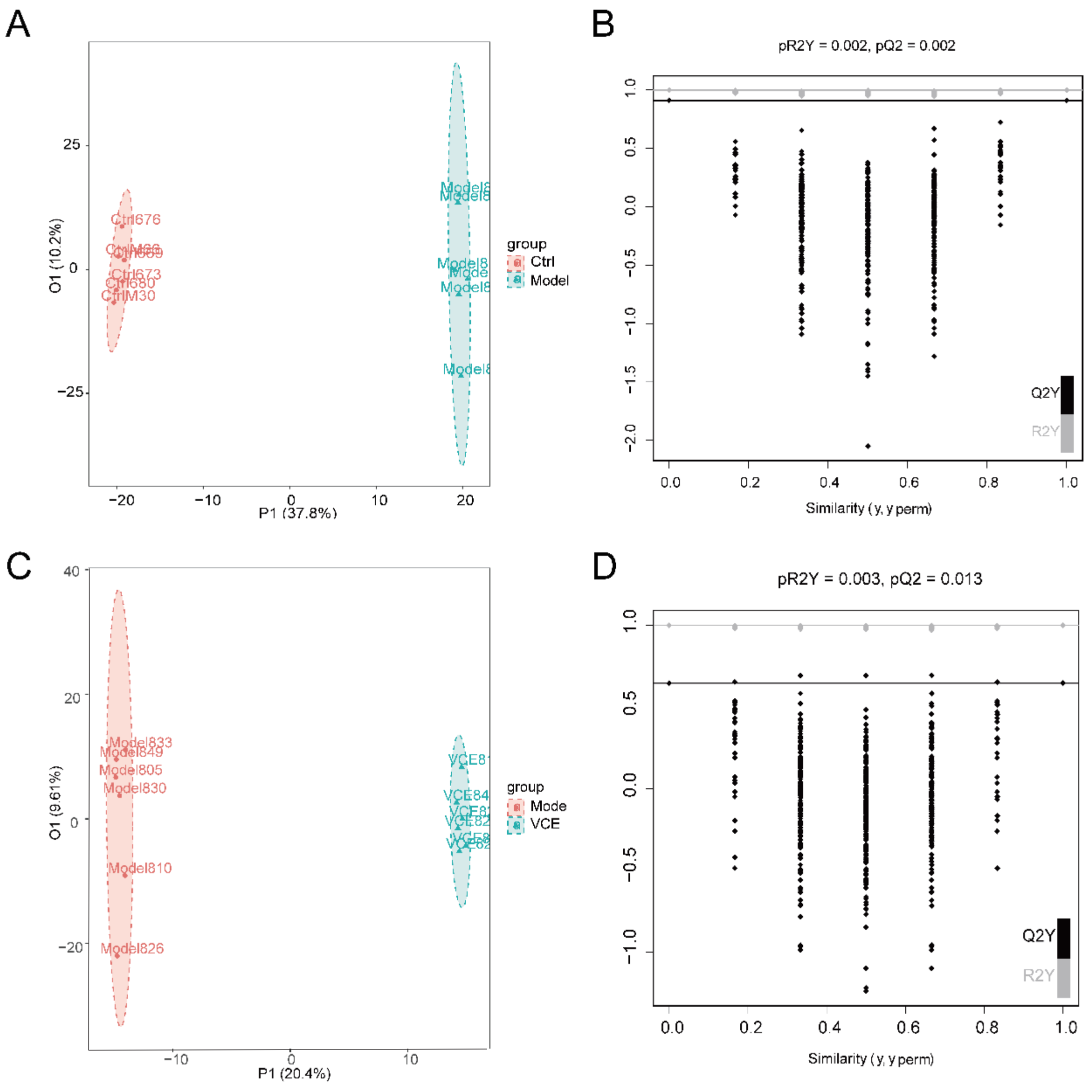

Figure S3 Analysis of PLS-DA and OPLS-DA. (A) Partial least squares discriminant analysis (PLS-DA) between normal and model groups. (B) Scatter plot of permutation test results of Orthogonal Partial Least Squares Discriminant Analysis (OPLS-DA) between normal and model groups. (C) Partial least squares discriminant analysis (PLS-DA) between model and VCE groups. (D) Scatter plot of permutation test results of Orthogonal Partial Least Squares Discriminant Analysis (OPLS-DA) between model and VCE groups.

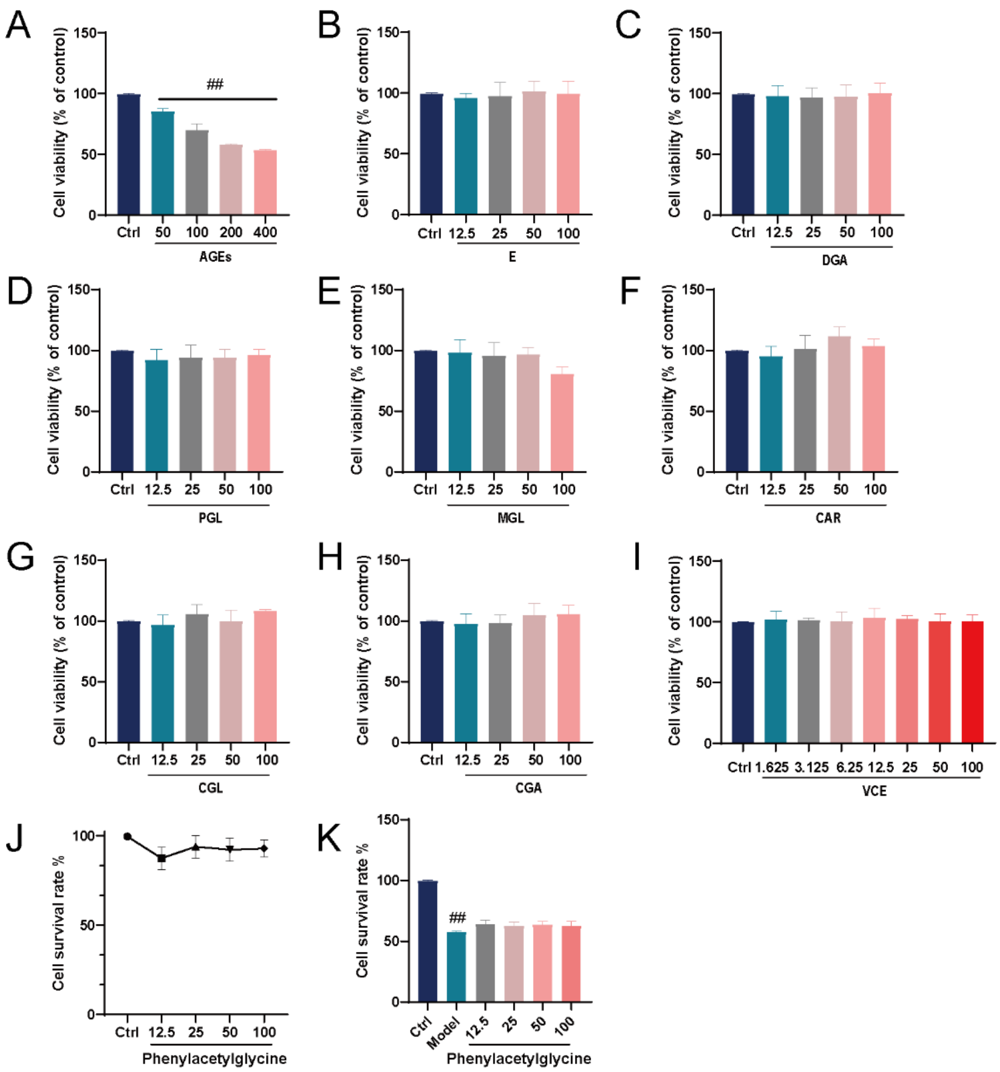

Figure S4 Cytotoxicity assay. Cytotoxicity assays of (A) AGEs, (B) Empagliflozin, (C) Delphinidin-3-galactoside chloride (DGA), (D) Petunidin-3-O-glucoside (PGL), (E) Malvin-3-O-glucoside (MGL), (F) Cyanidin-3-O-arabinoside (CAR), (G) Cyanidin-3-O-glucoside (CGL), (H) Cyanidin-3-O-galactoside (CGA), (I) Vaccinium myrtillus extract (VCE) and (J) Phenylacetylglycine. (K) Cell viability assay of phenylacetylglycine against AGEs-induced cell injury. Ctrl, control. Data are presented as the mean ± SD. #$p < 0.05$ and ##$p < 0.01$ vs. ctrl; *$p < 0.05$ and **$p < 0.01$ vs. model.

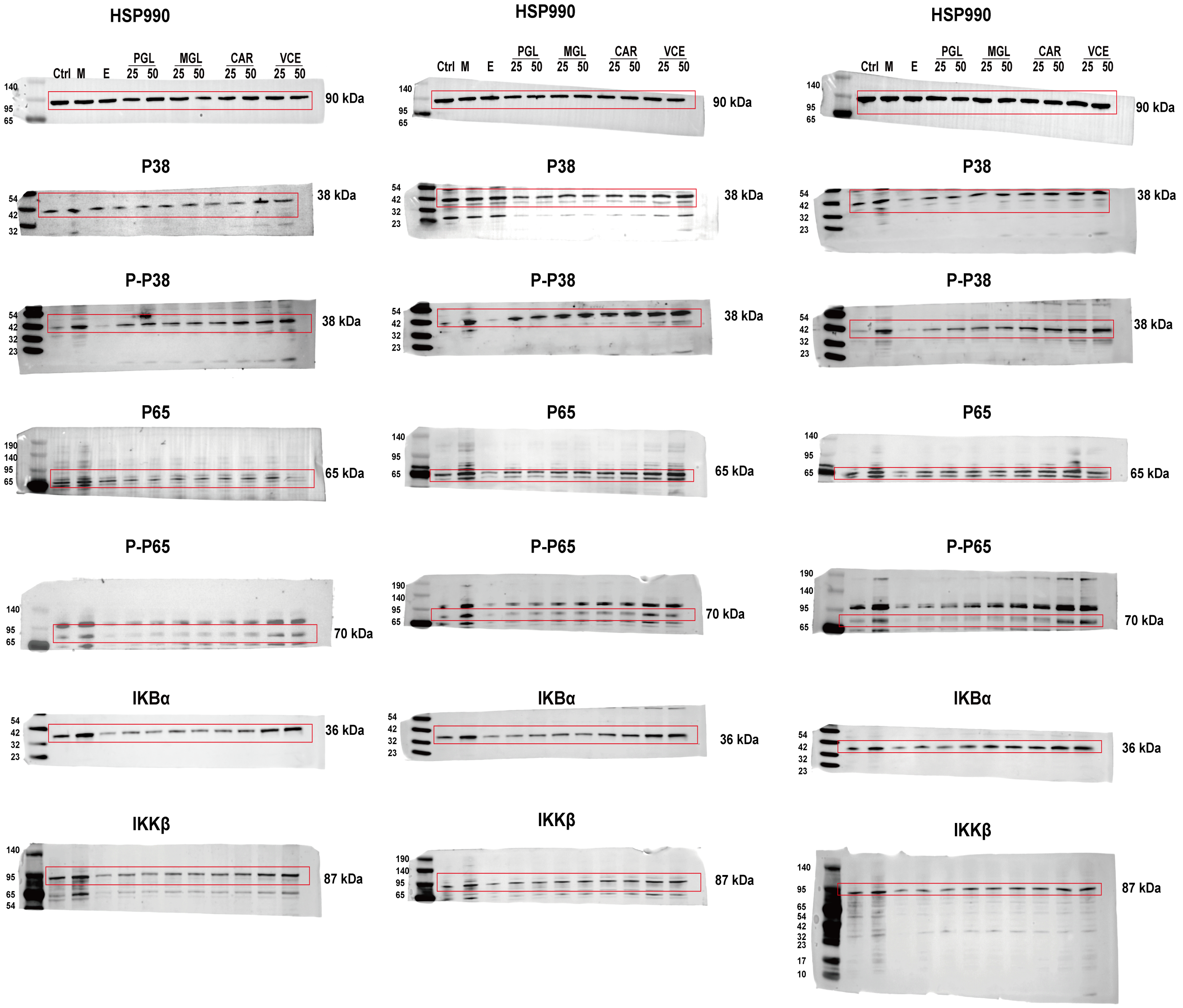

Figure S5 Western blot analysis of IKKβ, IκBα, p-p65, p65, p-p38, p38 and HSP90.
